# Supplementary material for: Immunophenotyping of a Stromal Vascular Fraction from Microfragmented Lipoaspirate Used in Osteoarthritis Cartilage Treatment and Its Lipoaspirate Counterpart
Source: Genes (Basel). 2019 Jun 21;10(6):474. doi: 10.3390/genes10060474 (PMC6627838; doi:10.3390/genes10060474)
Supplement: Supplementary file 1 [file genes-10-00474-s001.zip › Supplementary files/Figure S1.docx]

**LA MLA**


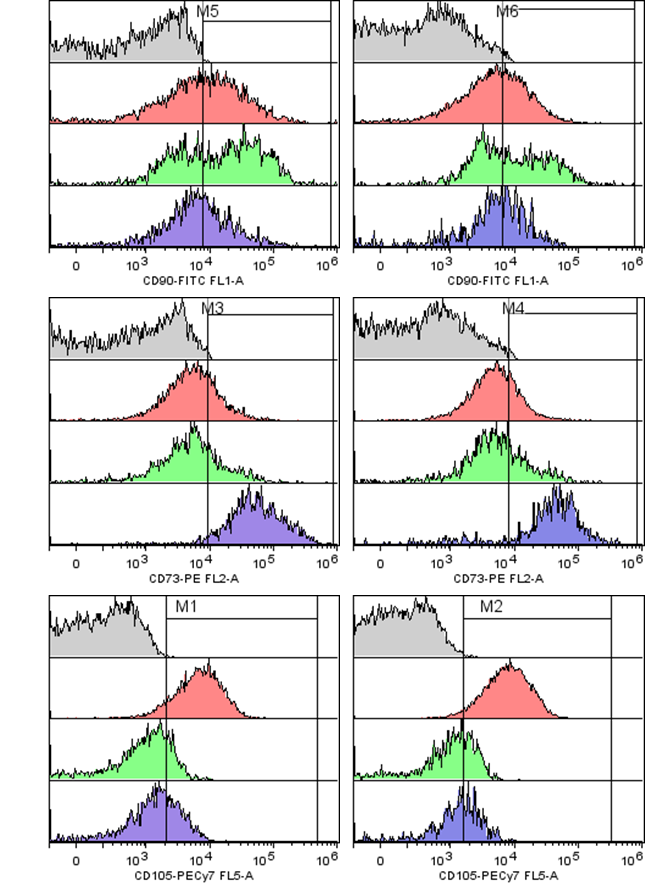


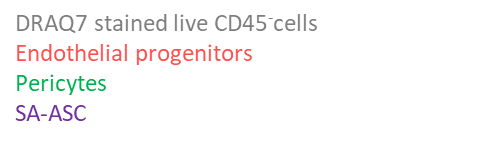


**Figure S1. Expression of MSC markers on endothelial progenitors, pericytes and SA-ASC.** Overlay oh histograms is used to show expression of CMSC marekrs CD90, CD73 and CD105 on endothelial progenitors (red histograms), pericytes (green histograms) and SA-ASC (blue histograms) on LA (left column) and MLA (right column). Shown are results from one representative sample out of the tvelve samples.. Signal from DRAQ7 only stained cells is shown with gray color.
