# Supplementary material for: Immunophenotyping of a Stromal Vascular Fraction from Microfragmented Lipoaspirate Used in Osteoarthritis Cartilage Treatment and Its Lipoaspirate Counterpart
Source: Genes (Basel). 2019 Jun 21;10(6):474. doi: 10.3390/genes10060474 (PMC6627838; doi:10.3390/genes10060474)
Supplement: Supplementary file 1 [file genes-10-00474-s001.zip › Supplementary files/Table S1.docx]

**Table S1. Reproducibility of staining with Duraclone SC Mesenchymal tube.**

Shown are representative overlays of tetraplicates of SVF cells isolated from LA of one patient and stained using Duraclone SC Mesenchymal tube. In total, 6 samples from different patients were stained, acquisited and analysed in the same manner. Data were analysed using Kaluza software (Beckman Coulter).

|  |
| --- |
|  |
|  |
|  |
|  |
|  |
|  |
|  |
