# Supplementary material for: Immunophenotyping of a Stromal Vascular Fraction from Microfragmented Lipoaspirate Used in Osteoarthritis Cartilage Treatment and Its Lipoaspirate Counterpart
Source: Genes (Basel). 2019 Jun 21;10(6):474. doi: 10.3390/genes10060474 (PMC6627838; doi:10.3390/genes10060474)
Supplement: Supplementary file 1 [file genes-10-00474-s001.zip › Supplementary files/Table S2_MIFlowCyt Item Checklist_Genes-507163.doc]

**Author Checklist: MIFlowCyt-Compliant Items**

| **Requirement** | **Please Include Requested Information** |
| --- | --- |
| 1.1. Purpose | The purpose of the study was to characterize stromal vascular fraction from microfragmented lipoaspirate (SVF-MLA) applied for cartillage treatment in OA and compare to that of autologous lipoaspirate (SVF-LA). |
| 1.2. Keywords | Lipoaspirate, microfragmented lipoaspirate, immunophenotyping, endothelial progenitors, pericytes, supra-adventitial adipose stromal cells. |
| 1.3. Experiment variables | Lipoaspirate treatment, gender. |
| 1.4. Organization name and address | Department for Translational Medicine, Srebrnjak Children's Hospital, Srebrnjak 100, Zagreb, Croatia |
| 1.5. Primary contact name and email address | Denis Polančec, dpolancec@bolnica-srebrnjak.hr |
| 1.6. Date or time period of experiment | Data were collected and analysed between December 2017 and December 2018. |
| 1.7. Conclusions | We performed a comparative polychromatic flow cytometry analysis of stromal vascular fraction (SVF) from collagenase-treated lipoaspirate (LA) and collagenase-treated microfragmented lipoaspirate (MLA). The immunophenotyping profile of SVF-MLA was dominated by reduction of leukocytes and SA-ASC and augmentation of EP, evidencing a marked enrichment of this cell population in the course of adipose tissue microfragmentation. In contrast to some previous papers that reported CD105 positivity on freshly isolated SVF-LA cells we did not find CD105 expression on pericytes and SA-ASC of either SVF-LA or SVF-MLA samples. The pericytes/SA-ASC ratio was significantly higher in SVF-MLA versus SVF-LA samples. Samples with the highest pericytes/SA-ASC ratio originate from male patients and this was significantly higher compared to female patients. The EP/pericytes and EP/SA-ASC ratios were also significantly higher in SVF-MLA compared to SVF-LA, however, it did not show sex-related difference in the former. |
| 1.8. Quality control measures | FlowCheck-pro beads and Flow-Set Pro beads were used daily for QC and to monitor sensitivity. BD Anti-Mouse Ig, κ/Negative Control Compensation Particles Set was used for the compensation. Unstained samples and DRAQ7-only stained samples were used for setting up gates. |
| 2.1.1.1. (2.1.2.1., 2.1.3.1.) Sample description | Human lipoaspirate and microfragmented lipoaspirate |
| 2.1.1.2. Biological sample source description | LA and MLA samples were obtained from twelve patients with osteoarthritis (6 males and 6 females, aged 30-81).The LA and MLA patient samples were delivered to the Srebrnjak Children's Hospital (Zagreb, Croatia) and stored overnight at room temperature (RT), protected from light before further processing. |
| 2.1.1.3. Biological sample source organism description | Homo sapiens, 6 males, 6 females |
| 2.1.2.2. Environmental sample location | N/A |
| 2.1.3. Other samples | Flow-Check Pro Fluorospheres (Beckman Coulter A63493)  Flow-Set pro Fluorospheres (Beckman Coulter A63492)  BD Anti-Mouse Ig, κ/Negative Control Compensation Particles Set (BD 552843) |
| 2.3. Sample treatment description | LA and MLA samples were digested with 1 % collagenase type I in D- MEM (both from Sigma-Aldrich, Saint Louis, MO, USA) in a shaking bath at 37°C for 45 minutes. After dilution 1:2 with 2% fetal bovine serum (Biosera, Nuaille, France) in D-MEM (Sigma-Aldrich), samples were filtrated through a 100 µm-cell strainer (BD Falcon, Corning, NY) and washed. Supernatants were discarded and cell pellet resuspended in 1 ml of the VersaLyse solution (Beckman Coulter, Miami, FL, USA). After 10 minutes, samples were filtered through a 40 µm-cell strainer (BD Falcon, Corning, NY), washed with D-MEM (Sigma-Aldrich) and cell pellet resuspended in D-MEM (Sigma-Aldrich). The cells were counted on the Sysmex XT1800 counter (Sysmex, Kobe, Japan). |
| 2.4. Fluorescence reagent(s) description |  |
| INSTRUMENT \| \| \| ANTIBODIES / DYES \| \| \| \|; LASER \| \| NAVIOS DETECTOR \| TARGET \| Clone \| FLUOROCHROME / DYE \| CATALOG # \| MANUFACTURER; 488nm \| FL1 \| \| CD90 \| F15_42 \| FITC \| DuraClone - prototype \| Beckman Coulter; \| FL2 \| \| CD73 \| AD2 \| PE \| DuraClone - prototype \| Beckman Coulter; \| FL3 \| \| CD34 \| 581 \| ECD \| DuraClone - prototype \| Beckman Coulter; \| FL4 \| \| CD146 \| TEA1/34 \| PC5.5 \| DuraClone - prototype \| Beckman Coulter; \| FL5 \| \| CD105 \| TEA3_17 \| PC7 \| DuraClone - prototype \| Beckman Coulter; 638 nm \| FL6 \| \| / \| / \| / \| / \| /; \| FL7 \| \| DNA \| / \| DRAQ7 \| B25595 \| BioStatus; \| FL8 \| \| CD45 \| J33 \| AA750 \| DuraClone - prototype \| Beckman Coulter; 405 nm \| FL9 \| \| CD31 \| 5_6E \| Pacific Blue \| DuraClone - prototype \| Beckman Coulter; \| FL10 \| \| Dead cells \| \| Live/Dead Fixable Yellow \| L34959 \| ThermoFisher | |
| 3.1. Instrument manufacturer | Beckman Coulter |
| 3.2. Instrument model | Navios |
| 3.3. Instrument configuration and settings | The flow cytometer Navios has been configured by the manufacturer. For the set up of the instrument “EuroFlow SOP for Instrument set-up and compensation for Navios Instruments Version 1.4 was used (https://www.euroflow.org/usr/pub/protocols.php). |
| 3.3.1. Flow Cell and Fluidics | Sensing area: BioSense 150 µm x 460 µm rectangular cuvette. Flow-Check Pro and Flow-Set Pro beads were acquired at approximately 10 µl/min. Compensation beads, LA and MLA samples were acquired at approximately 60 µL/min. |
| 3.3.2. Light sources | Navios is equipped with three solid-state, software controlled lasers.  1) 22 mW, blue laser operating at 488 nm, elliptical beam spot 10 µm x 84 µm  2) 25 mW, diode laser operating at 638 nm, elliptical beam spot 9.6 µm x 72 µm  3) 40 mW, violet laser operating at 405 nm, elliptical beam spot 8.9 µm x 70 µm |
| 3.3.3. Optical fliters | Optical configuration and transmitted wavelengths are show at diagram bellow (diagram was taken from “Navios Flow Cytometer: Instructions for Use” manual that was supplied by Beckman Coulter at the time of instrument pucrchase during the year 2012.   |
| 3.3.4. Optical detetors |  |
| 3.3.4.1. Optical detector types, voltages and amplification | Data about detector and instrument settings are listed in table below: |
|  | |

| Laser | Detector | Detector type | Parameter / Fluorochrome | Target channel value (MFI)  for Flow-Set Pro Beads | Voltage settings | Amplification |
| --- | --- | --- | --- | --- | --- | --- |
| 488 nm | Wide | Segmented photodiode | FSC Intensity and  FSC Time of Flight | / |  | Linear |
|  | / | Photodiode | SSC Intensity and  SSC Time of Flight | / |  | Linear |
|  | FL1 | PMT | FITC | 68-76 | 500 | Log |
|  | FL2 | PMT | PE | 232-262 | 500 | Log |
|  | FL3 | PMT | ECD | 146-166 | 425 | Log |
|  | FL4 | PMT | PC5..5 | 116-124 | 550 | Log |
|  | FL5 | PMT | PC7 | 54-58 | 570 | Log |
| 638 nm | FL6 | PMT | APC | 15-17 | 485 | Log |
|  | FL7 | PMT | DRAQ7 | 260-296 | 450 | Log |
|  | FL8 | PMT | AA750 | 73-83 | 485 | Log |
| 405 nm | FL9 | PMT | Pacific Blue | 55-61 | 425 | Log |
|  | FL10 | PMT | Live/Dead Yelow | 110-124 | 348 | Log |

| 4.2. Compensation description | Tubes with single dried DuraClone reagent were used for compensation samples as follow: CD4 FITC, CD4 PE, CD34 ECD, CD146 PE-Cy5.5, CD105 PE-Cy7, CD45 APC-AlexaFluor750 and CD8 Pacific Blue. FACSComp beads, Negative control and Anti-Mouse IgG beads, were added to each tube. Then 100 µl of PBS was added, tubes briefly vortexed and proceeded further in the same manner as the DuraClone SC tubes. For the DRAQ7 compensation mixture of DRAQ7 stained and unstained SVF cells was used. Compensation was not performed for the Live/Dead Fixable Yellow stain because only negative cells were used in analysis. |
| --- | --- |
| 4.3. Data transformation details | N/A |
| 4.4.1. Gate description | Gating procedures applied was, with minor modifications, as described in articles:  Zimmerlin, L.; Donnenberg, V.S.; Pfeifer, M.E.; Meyer, E.M.; Peault, B.; Rubin, J.P.; Donnenberg, A.D. Stromal vascular progenitors in adult human adipose tissue. *Cytometry. Part A : the journal of the International Society for Analytical Cytology* **2010**, *77*, 22-30.  Zimmerlin, L.; Donnenberg, V.S.; Rubin, J.P.; Donnenberg, A.D. Mesenchymal markers on human adipose stem/progenitor cells. *Cytometry. Part A : the journal of the International Society for Analytical Cytology* **2013**, *83*, 134-140.  Because of unavailability of the FMO controls, SVF cells were stained with the DRAQ7 dye only, and then regions and gates set as described in article.  The gating tree was set as follows (as shown on Figures :   1. FSC LIN/FSC TOF: „Singlets“: exclusion of doublets, to 2. SSC/DRAQ7: „Nucleated cells“: exclusion of debris and selection of nucleated cells, to 3. DRAQ7/CD45 APC-AlexaFluor750: Selection of CD45^-^ cells, to 4. CD34 ECD/CD31 Pacific Blue: determination of CD31^+^CD34^-^ endothelial mature cells, determinatin of CD31^+^CD34^+^ endothelial progenitor cells and selection of CD31^-^ cells for further analysis, to 5. CD34 ECD/CD146 PC5.5: determination of CD34^-^CD146^+^ pericytes, CD34^+^CD146^+^ transitional pericytes and CD34^+^CD146^-^ supra-adventitial adipose stromal cells.   6. Quadrants were set for analysis of the expression of CD73, CD90 and CD105 surface  markers on three dominating supopulations – endothelial progenitors, pericytes and SA-ASC.. Since for those markers CD45- SVF cells doesn’t have clear bimodal expression, quadrants were positioned on a basis of DRAQ7 stained SVF cells. |
| 4.4.2. Gate statistics | Percentage positive from live nucleated events were determined for CD45+ cells, endothelial progenitor cells, endothelial mature cells, pericytes, transitional pericytes and SA-ASC. |
| 4.4.3. Gate boundaries | Regions and gates were positioned with the aid of DRAQ7-only stained sample and according to gating strategies published in articles listed under the 4.4.1. section. Batch processing of the FCS data analisys was performed for all tvelve samples and regions and gates minimaly adjusted were needed. |
